# Surrounding-aware representation prediction in Birds-Eye-View using transformers

**DOI:** 10.3389/fnins.2023.1219363

**Published:** 2023-07-04

**Authors:** Jiahui Yu, Wenli Zheng, Yongquan Chen, Yutong Zhang, Rui Huang

**Affiliations:** ^1^Shenzhen Institute of Artificial Intelligence and Robotics for Society, and the SSE/IRIM, The Chinese University of Hong Kong, Shenzhen, Guangdong, China; ^2^The Shenzhen Academy of Inspection Quarantine, Shenzhen, Guangdong, China

**Keywords:** BEV maps, deep learning, attention, transformers, autonomous driving

## Abstract

Birds-Eye-View (BEV) maps provide an accurate representation of sensory cues present in the surroundings, including dynamic and static elements. Generating a semantic representation of BEV maps can be a challenging task since it relies on object detection and image segmentation. Recent studies have developed Convolutional Neural networks (CNNs) to tackle the underlying challenge. However, current CNN-based models encounter a bottleneck in perceiving subtle nuances of information due to their limited capacity, which constrains the efficiency and accuracy of representation prediction, especially for multi-scale and multi-class elements. To address this issue, we propose novel neural networks for BEV semantic representation prediction that are built upon Transformers without convolution layers in a significantly different way from existing pure CNNs and hybrid architectures that merge CNNs and Transformers. Given a sequence of image frames as input, the proposed neural networks can directly output the BEV maps with per-class probabilities in end-to-end forecasting. The core innovations of the current study contain (1) a new pixel generation method powered by Transformers, (2) a novel algorithm for image-to-BEV transformation, and (3) a novel network for image feature extraction using attention mechanisms. We evaluate the proposed Models performance on two challenging benchmarks, the NuScenes dataset and the Argoverse 3D dataset, and compare it with state-of-the-art methods. Results show that the proposed model outperforms CNNs, achieving a relative improvement of 2.4 and 5.2% on the NuScenes and Argoverse 3D datasets, respectively.

## 1. Introduction

The advancement in deep learning has facilitated a better understanding of semantic representation and contributed to more accurate prediction of object locations. This line of research has a wide range of applications in autonomous driving (Ohn-Bar et al., 2020; Yi et al., 2021; Cao et al., 2022a; Wang et al., 2022).

**Figure 1:**
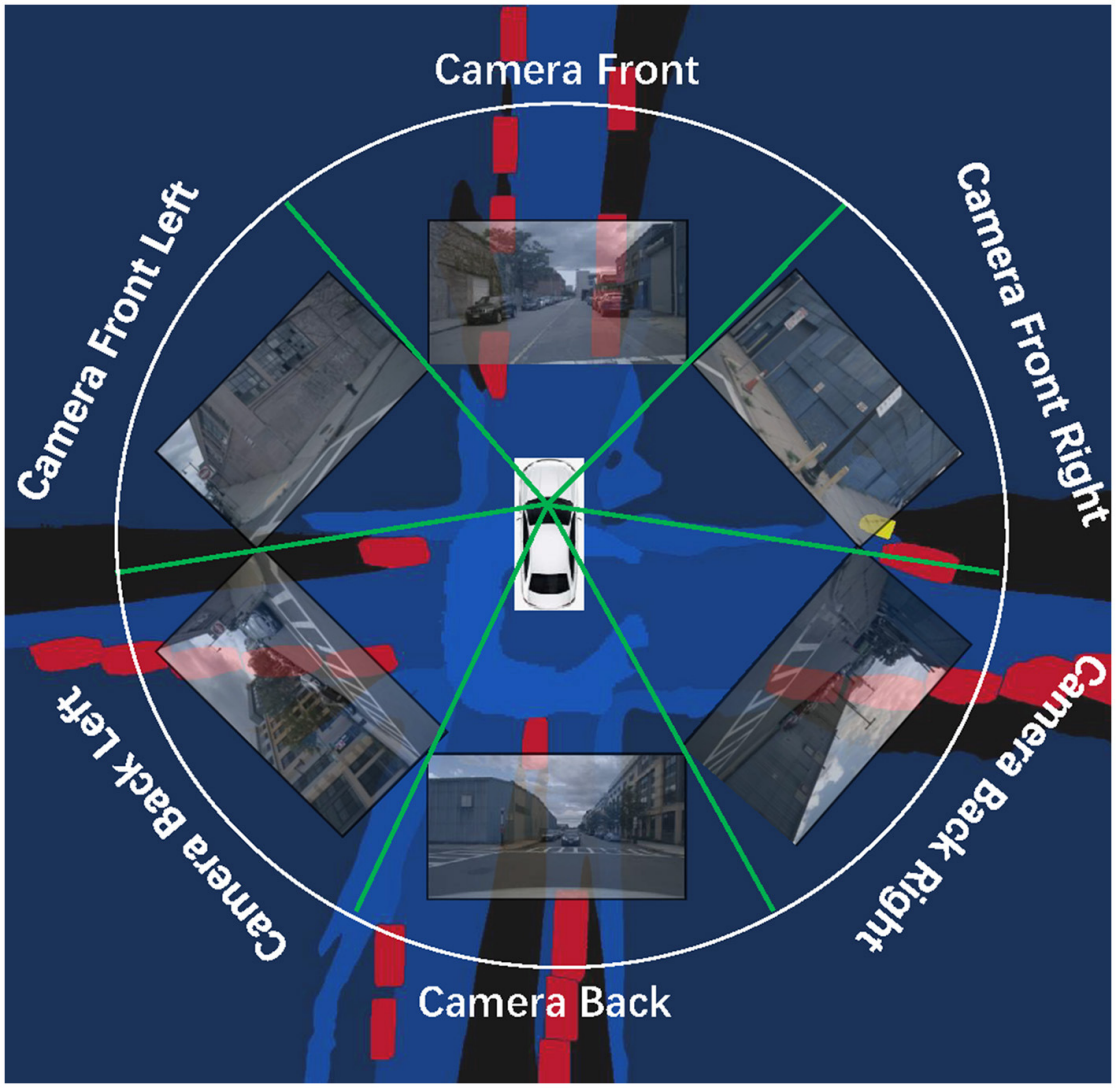
Schematic illustrating the process of BEV semantic representation prediction in environment perception. A set of surround-view monocular cameras are used to capture the surrounding environment (RGB images). These images are passed through a road-aware system to generate BEV maps containing the location and shape information of various elements.

Recent studies have made significant strides in mapping multiple side-view images to Birds-Eye-View (BEV) semantic maps, aiming to predict the positional probability of each element. These BEV maps have proven to be potent tools for environment perception, fundamental to autonomous navigation and driver assistance systems. As illustrated in Figure 1, cameras strategically positioned around the vehicle capture RGB images from all directions. Surrounding-aware systems then model these images to generate comprehensive 360-degree BEV maps, offering a panoramic understanding of the vehicle's environment. However, creating BEV maps is challenging; it represents a complex, multi-stage processing flow encompassing ground plane estimation, road segmentation, lane detection, and object detection, as described in Chen et al. (2020), Pan et al. (2020), and Roddick and Cipolla (2020). It's a laborious process with challenges, yet its importance for safe and efficient autonomous navigation cannot be overstated. The ideal scenario is to design an end-to-end framework powered by deep learning. This approach would directly predict the desired map representation from sensor observations, providing a comprehensive understanding of the environment in a single step. In this context, semantic segmentation emerges as an indispensable tool, particularly in autonomous driving. Semantic segmentation helps distinguish various environmental elements, like roads, pedestrians, vehicles, etc., enabling the system to interpret and interact safely with its surroundings. By integrating this with our proposed end-to-end BEV map generation, we aim to facilitate a more robust, efficient, and safer autonomous driving system.

Several studies, such as Hendy et al. (2020), Mani et al. (2020), Wu et al. (2021), Cao et al. (2022b), and Han et al. (2022), have shown that CNNs are capable of capturing a large receptive field; however, this comes with a trade-off involving deepening the neural network structure. Despite being highly discriminative, semantic features extracted from deeper convolution layers are not suitable for representing small-sized/multi-class elements, which limits the accuracy of predicting multi-element BEV representations. Recent studies, including Yi et al. (2021) and Yu et al. (2021), have indicated that shallow feature maps are more effective for small-scale object detection as they provide rich spatial information. As a result, balancing the need for capturing large receptive field and extracting highly discriminative features can be challenging for CNNs. Current studies have shown that Transformers are able to achieve feature extraction with a large receptive field in a shallow structure.

Exploring various strategies for developing high-quality Bird's Eye View (BEV) maps has become increasingly essential in technology and science, particularly with the rise of autonomous navigation and robotics. While several methodologies have been presented, they tend to rely on large training samples and display less resilience when faced with varying circumstances. Furthermore, these previous studies primarily utilized Transformers for tasks involving classification, which output a set of per-class probabilities as exhibited by Han et al. (2022), Hu et al. (2022), and Li et al. (2022). This leaves a significant area within the transformative potential of Transformers untapped—generating BEV semantic representation. We venture into relatively uncharted territory, exploring the potential of using Transformers exclusively to generate a BEV semantic representation, thereby bypassing the necessity for convolution layers. Unlike the conventional approaches, which focus on “classifying” image-based data, our approach looks at both input and output as images—a procedure we refer to as “image generation.” This shift from classification tasks to generation tasks, utilizing Transformers, might pave the way to more efficient, scalable, and diverse applications, ultimately expanding the possibilities of BEV mapping technologies. Comprehensive semantic feature extraction is the bedrock for constructing high-quality BEV maps. To improve this process, the research community must be willing to test novel approaches. Our proposed use of Transformers as a sole agent for generating BEV semantic representation stands as a pioneering endeavor in this domain, challenging the conventional paradigms that have been established. As such, it will contribute to the broader discourse on effective BEV mapping and extend the functional capabilities of Transformers. The outcomes of this study could potentially guide further developments and improvements in autonomous navigation systems, robotics, and other related areas where BEV maps are paramount. This research is not merely a theoretical experiment but a concrete step forward in the practical application of Transformers in the real world.

To achieve this goal, the paper addresses two main challenges: (1) how to extract global-local discriminative features using Transformers, and (2) how to generate pixels from image features without the use of convolution layers. A main challenge for Transformers is their high dependence on data availability for training the model to achieve promising performance. Additionally, the way attention-a core component of Transformers-is applied to image generation is still under-explored. In the paper, we propose a Transformer-based framework that generates BEV semantic representations in an end-to-end process. To fully capture features, a new attention mechanism is employed for spatial relationships. Traditional neural networks always overlook a large number of semantic features due to the projections of features in different planes. To overcome this limitation, we design a new plane transformation algorithm. Previous methods have relied mostly on convolution layers for multi-class representation generation. However, since Transformers and convolution would restrict the performance improvement of each other, a pure Transformer-powered generator is proposed to address the issue. Alongside this, a stable training scheme is also developed to specifically target the new framework.

The contributions of this study can be summarized as follows.

- We propose a new framework purely powered by Transformers for predicting BEV semantic representations. The framework achieves this objective in an end-to-end manner without convolution layers. This approach differs dramatically from the existing CNN-based methods, greatly simplifying the pipeline and improving perceptual details.
- We propose a feature extractor based on a Transformer to memorize global clues and further mine local clues. The extractor contains an overlapping patch generation method and multi-head attention-based blocks.
- We design a new generator powered by Transformers to generate pixels based on per-class prediction probabilities. The carefully-designed pipeline is essential for generating successful BEV semantic representations. In addition, a simple trick is proposed that can transform image features into BEV features.
- We achieve competitive results on two challenging datasets, namely 19.9% Mean IoU on the NuScenes dataset and 19.1% Mean IoU on the Argoverse 3D dataset. Compared with leading CNN-based methods, our model demonstrate an improvement of 2%–6 % in Mean IoU, and about 1% IoU improvement is achieved on challenging prediction tasks.

The remainder of the paper is structured as follows. Section 2 reviews deep learning-powered studies. Section 3 provides details on the proposed frameworks and technologies. Section 4 presents the experimental results and discussion. Section 5 concludes the works and shows the future research direction.

## 2. Related work and basics

### 2.1. Surrounding-aware system in autonomous driving

The surrounding-aware system plays a crucial role in connecting road conditions and driving assistance systems. However, current technologies, such as sensing, detection, and segmentation, are limiting the development of these systems Predicting BEV representation based on monocular images is a challenging problem for several reasons including multitasking, complex 3D estimation, and multi-class prediction. Traditional studies have proposed neural networks based on semantic segmentation for BEV representation prediction (Pan et al., 2020; Lu et al., 2021). However, these 2D representations lack spatial relationships and are not effective in complex 3D spatial scenarios. Recent studies have reported two categories of solving this challenge: camera geometry and transformation implicitly, as reported by Lu et al. (2021) and Yao et al. (2021). The former has achieved significant performance by using on multi-type data input, while the latter is more suitable for building a simple learning framework in an end-to-end manner.

Although current methods are effective based on single data input, they are not able to fully mine the spatial dependency, which severely hinders the performance of multi-scale/multi-class element prediction. In the paper, the proposed neural networks are specifically developed to exploit rich spatial clues by considering the global spatial relationship in a shallow framework, instead of focusing on particular regions in a deep structure. The purely Transformer-based neural networks are proposed in the paper, and several related or derived techniques are developed.

### 2.2. CNN-based studies

**Figure 2:**
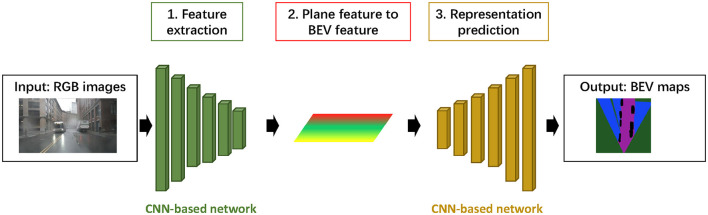
Architecture of CNN-based networks for BEV representation prediction.

In previous studies, Convolutional Neural networks (CNN) have been widely used for image processing. As shown in Figure 2, the process typically contains three steps: (1) extracting features from input images, (2) projecting planar image features to BEV features, and (3) generating pixels under the BEV map. Current studies have utilized leading CNN-based backbones for feature extraction, such as ResNet (He et al., 2016), Feature Pyramid Network (FPN) (Lin et al., 2017), and DeepLab network (Yang et al., 2018). In addition, recent works by Hendy et al. (2020) and Mani et al. (2020), have incorporated BEV view transformation based on FPN and employed adversarial loss to optimize BEV representation. Inspired by the Generative Adversarial Network (GAN), some studies have proposed Top-down networks, with the Inverse Perspective Mapping (IMP). The front view image is mapped to the ground plane by homography (Zhu et al., 2018; Hu et al., 2023).

However, a significant issue has arisen in these CNN-based studies. While the accurate prediction of large-size objects has reached a saturation point, the forecast of small-size things still remains an unresolved challenge. The majority of CNN-based networks excel in local semantic segmentation. Still, they need to catch up when predicting global BEV maps. The reason for this lies in the inherent trade-off between network depth and the range of the receptive field. In CNN-based networks, the receptive field can access the depth of the network. While this sounds beneficial in theory, allowing the network to capture more complex patterns with more layers, it also escalates the challenges associated with model training. Deeper networks tend to suffer from difficulties in training due to issues such as vanishing and exploding gradients. Moreover, as the web grows in depth, it becomes increasingly computationally intensive, which might not be sustainable in real-world, resource-limited applications. In light of this, there is a pressing need for novel solutions that can accurately predict large and small objects in BEV maps while also addressing the computational and training challenges associated with deep CNN-based networks. We can only unlock the full potential of BEV mapping for autonomous navigation and related applications by overcoming these hurdles.

### 2.3. Transformer-based studies

In 2020, Google AI introduced Vision Transformer (ViT) for image classification without convolution layers (Dosovitskiy et al., 2020). ViT divides the input image into square patches of equal sizes, followed by the pure Transformer architecture processing directly on the patch sequence to mine global-local features and output per-class probabilities. Originally, Transformers were proposed for Natural Language Processing (NLP) tasks (Vaswani et al., 2017), but ViT has achieved impressive performance on multiple image recognition benchmarks. Transformers have also been used to solve other vision-related problems, including object detection, semantic segmentation, and image processing, where they outperformed CNN-based networks including object detection, semantic segmentation, and image processing (Han et al., 2022). Typical studies by Carion et al. (2020), Chen et al. (2021a), Misra et al. (2021), and Mazzia et al. (2022), have reported that self-attention mechanism used in Transformers help model long-term features effectively. Furthermore, some studies, such as Ba et al. (2016), Liu et al. (2021), and Zheng et al. (2021) have extended Transformers to the field of semantic segmentation by designing the encoder based on Transformers, then adding other existing decoders to model the image context. However, the following limitations exist in recent Transformers-based studies: (1) the global modeling scheme leads to high computational costs and requires a large amount of data, and (2) the decoder design still relies on convolutional layers. To the best of our knowledge, no prior studies have explored a pure Transformer-based framework for predicting BEV semantic representations without convolution layers.

## 3. Method

**Figure 3:**
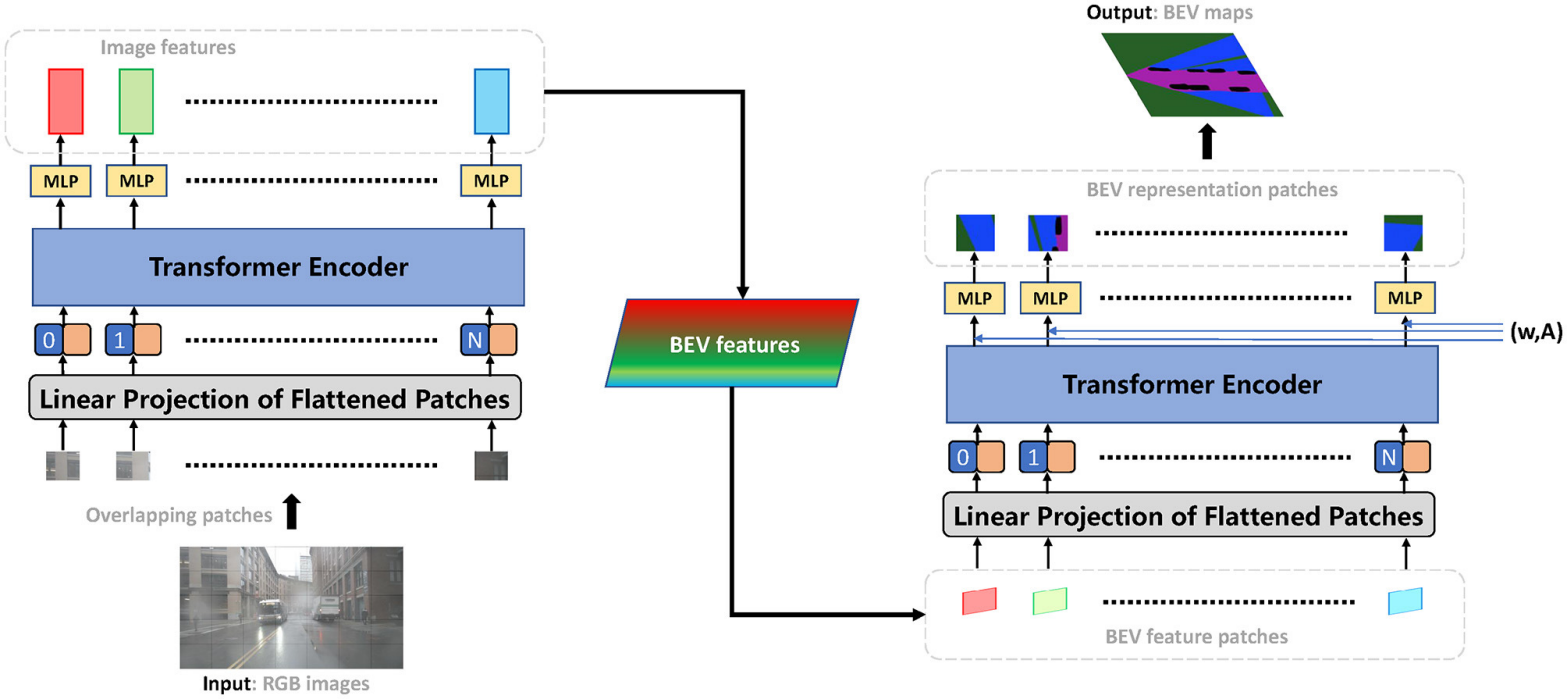
Architecture of the proposed approach. The end-to-end framework contains three modules: (1) Feature extraction from input images, (2) Image features are projected to BEV features, and (3) BEV semantic representation prediction. A Transformer-based network encodes global-local spatial features powered by multi-head attention. The image-plane features can be transformed to BEV-plane features with the smallest possible loss of feature information. A generative network further processes BEV global-local spatial features and predicts the final classification probabilities.

We approach the prediction of BEV representation by framing it as a global-local spatial relationship mining problem. Given a set of look-around image inputs, the proposed method generates the corresponding BEV representations in order. These representations can be simply synthesized into full-space BEV maps. In this section, we present the technical details of our proposed method according to the processing order in the end-to-end framework, as shown in Figure 3. We begin by explaining a Transformer-based extractor that achieves image encoding, while balancing the global spatial attention and receptive field. Next, we describe how we transform the side views into BEV views and mine the relationship of the inter-frame through a homography-based algorithm. We also present a new Transformer-based predictor for making predictions in BEV views. To represent the state of the world, including vehicles, drivable areas, and land boundaries, we use the semantic occupancy grid, which is an extension of occupancy grid maps. We then introduce an association training scheme that ensures the stable convergence of Transformer-based neural networks.

### 3.1. Image encoding

In alignment with the conventional Vision Transformer (ViT) method, our proposed model uses a backbone to extract image features crucial for generating Bird's Eye View (BEV) semantic representations. The extraction process is mathematically outlined in Equation (1), with the details underpinning this method comprehensively discussed in Dosovitskiy et al. (2020). Our feature extractor incorporates several vital components: multi-head self-attention, multilayer perceptrons (MLPs), residual connections, layer normalization, positional encoding, and meticulously structured network topology. Each component plays an instrumental role in the overall process of BEV generation. The multi-head self-attention mechanism is particularly crucial in this process. It enables the model to focus on different parts of the input image simultaneously, thus allowing it to capture complex patterns and dependencies in the input data. This capability is vital for tasks like BEV prediction, where various aspects of an image contribute to the final output. Multilayer perceptrons further enhance the model's capability to understand complex patterns in the data. At the same time, residual connections help combat the vanishing gradient problem, enabling the model to learn more effectively from the data. Layer normalization ensures that the model's training remains stable and efficient by standardizing the inputs to each network layer. Meanwhile, positional encoding is employed to provide the model with information about the relative positions of the pixels in the input image, which is crucial for tasks involving spatial data. Finally, the network topology defines the overall structure of the model and is designed in a way to optimize the information flow and processing within the model. By intertwining these components, our feature extractor presents an effective means of obtaining and interpreting image data, fulfilling the essential role in BEV generation.


(1) $$\begin{array}{l} z_{0}=[x_{class};x_{p}^{1}E;x_{p}^{2}E;......;x_{p}^{N}E]+E_{pos},\\ E\in R^{(P^{2}\cdot C)\times D},E_{pos}\in R^{(N+1)\times D}\\ z_{\ell }{}^{\prime }=MSA(LN(z_{\ell -1}))+z_{\ell -1},\ell =1......L\\ z_{\ell }=MLP(LN(z_{\ell }{}^{\prime }))+z_{\ell }{}^{\prime },\ell =1......L \end{array}$$

For image encoding, we design a Transformer-based network considering the requirements of feature transformation. First, the input of the network *X* takes an image with three dimensions (*C*×*H*×*W*) as input and converts it into a 2D vector [$x_{p}\in HW/P^{2}\times \left(P^{2}\cdot C\right)$]. To achieve the conversion, we split the image into multiple patches of fixed-size (size: *P*×*P*) that is carefully designed to mine local cues more effectively. Specifically, each patch is extended by *i* pixels to create overlap between patches, so the size of each patch is (*P*+2*i*) × (*P*+2*i*). Our research has shown that this careful design achieves regular training and robust optimization. Moreover, the embedding matrix *E*can covert each patch to the (*N*×*D*) dimensions, and the *E*_*pos*_ is the position code to prevent the patch order from being disrupted. MSA means the operation powered by the multi-head self-attention (MSA) whose technical details are similar to that of the ViT. The MLP consists of the Linear layer (LN) and the tanh function.

Due to poor inductive bias performance, Transformer-based models show high sensitivity to input perturbations. However, to enhance their generalization performance, the proposed neural networks are expected to be insensitive to input perturbations. To achieve this, a new attention (*A*_*E*d_(*X*)) is introduced in the transformer encoder process. This new attention model draws inspiration from the contributions in Kim et al. (2021), and recomputes the dot product similarity in attention using Euclidean distance, as illustrated in Equation (2), where *P*_*q*_, *P*_*k*_, and *P*_*v*_ respectively denote the important parameters in the projection process i.e., Query, Key, and Value; and *d*_*n*_ denotes the dimension of features in multi-head attention.


(2) $$\begin{array}{l} A_{E\text{d}}\left(X\right)=Soft\max\left(Ed\left(XP_{q},XP_{k}\right)/\sqrt{d_{n}}\right)XP_{v} \end{array}$$

Our proposed approach diverges significantly from typical Transformer-based classification models in terms of its output. In conventional models, the multilayer perceptrons (MLPs) output within the Transformer is typically a set of classification probabilities. However, in our work, the MLPs output image features. These features, rich in semantic information, are then transformed into Bird's Eye View (BEV) features, as further detailed in the following sub-section. This innovation has multiple potential benefits. Most importantly, it has a considerable impact on the computational efficiency of our model. Given that image features contain essential information in a condensed form, this method dramatically reduces the volume of data to be processed in subsequent stages. Instead of classification probabilities, the output features bring the significant advantage of lowering the model's complexity and reducing the computation load. Furthermore, the model can bypass the computationally intensive step of converting probabilities back into image features by directly working with image features instead of possibilities. This further economizes the computational resources required, making the model more efficient and quicker. In essence, our approach is designed to effectively extract and utilize image features for BEV mapping, all while maintaining computational efficiency. This streamlined process, which provides detailed BEV features without the usual computational burdens, is a key advancement over traditional Transformer-based classification models.

### 3.2. Image-to-BEV

The process of converting a side view captured by the vehicle camera to the BEV perspective is significantly challenging, largely due to the fundamental differences between the two coordinate systems. Unfortunately, the feature extraction network can only output image-plane features. Hence, the main objective is to reduce feature loss during the feature transformation process. An image-plane feature map that has a height *H* and width *W* is transformed into a BEV-plane that has a depth *Z* and width *X*, with channel *C* unchanged.

Motived by the Hough transform, we design an effective method for projecting features from image-plane to BEV-plane features (*F*^*IP*^→*F*^*BEV*^), as shown in Equation (3). Where *r*_*l*_, θ_*l*_, and *c*_*l*_ denote horizontal plane, azimuth, and elevation in a feature map location, respectively; and *w*_(_*r*__*l*_, θ_*l*_)_ denotes the weights learned by the framework. Technically, we first collapse the vertical and channel dimensions into a transition dimension and keep the horizontal dimension unchanged. We then reset the transition features to get a new tensor with the size (*C*×*Z*×*W*). Finally, we resample into a Cartesian coordinate system, namely, the BEV of the trapezoid, thus establishing a new camera geometry.


(3) $$F_{\left(r_{l},\theta_{l},c_{l}\right)}^{BEV}=\sum w_{\left(r_{l},\theta_{l}\right)}\times F_{\left(r_{l},\theta_{l},c_{l}\right)}^{IP}$$

Compared with recent studies, such as Pan et al. (2020) and Philion and Fidler (2020), the proposed method utilizes a cost-saving operation to address the challenge of retaining the depth features of the input.

### 3.3. BEV semantic representation generation

Generating semantic features entirely using Transformer-based model can be a huge challenge because the Transformers need to generate pixels in spatial regions instead of traditional predicted class labels. Inspired by studies of combining Transformers to GANs, such as Chen et al. (2021b), Jiang et al. (2021), an Lee et al. (2021), we proposed a new projector to generate BEV semantic representation without convolution and pooling layers through two stages: BEV semantic representation generation from single image and BEV representation fusion. Unlike GANs, the discriminator (a special Loss of GAN) is not required, and we design an associate training scheme to supervise the end-to-end framework.

We develop the Transformer Encoder module to generate new pixels in spatial space. We first introduce an affine transformation A to each image feature patch, followed by the use of the Fourier function for patch embedding. In technical terms, the architecture is represented by Equations (4) and (5), where (*x, y*) denotes the values of patch pixel obtained from the patch embedding, *L* is the length of the input sequence, *E*_*fou*_ is the Fourier encoding to compute the spatial position of the pixel, and *M*_θ_ is the MLP operation. The results show that the proposed module is effective in generating BEV pixels, as shown in the details presented in Figure 6.


(4) $$\begin{array}{l} z_{0}=E_{pos},E_{pos}\in R^{(N+1)\times D}\\ z_{\ell }{}^{\prime }=MSA(LN(z_{\ell -1},w))+z_{\ell -1},\ell =1......L\\ z_{\ell }=MLP(LN(z_{\ell }{}^{\prime },w))+z_{\ell }{}^{\prime },\ell =1......L \end{array}$$

(5) $$\begin{array}{l} y=LN(h_{L},w)\\ x=[M_{\theta }(E_{fou},y^{1}),......,M_{\theta }(E_{fou},y^{L})] \end{array}$$

Motivated by the multiple observation information methods (Wang et al., 2019; Roddick and Cipolla, 2020), we propose a Bayesian-based information natural fusion method. The main objective is to build a wraparound BEV representation by calculating the occupancy probability of each view feature in the global coordinate system. First, we use the Log-odds operation (denoted as $l_{i,t}^{c}$) to equate the occupancy probability $p\left(m_{i}^{c}|o_{t}\right)$ that are the output of the network, where $m_{i}^{c}$ is the *i*-th observation of an object of class *c*in network output. Next, The combination of observations from the 1st to the *t*-th is shown in Equations (6) and (7).


(6) $$\begin{array}{l} l_{i,1:t}^{c}=l_{i,1:t-1}^{c}+l_{i,t}^{c}-l_{0}^{c}\\ l_{0}^{c}=\frac{p(m_{i}^{c})}{1-p(m_{i}^{c})} \end{array}$$

(7) $$l_{0}^{c}=\frac{p(m_{i}^{c})}{1-p(m_{i}^{c})}$$

### 3.4. Training

We design an association training scheme to obtain more accurate predicted probabilities containing three Loss functions, as shown in Equation (8). First, the Binary cross-entropy (*L*_*bce*_) is used to train semantic occupancy probabilities *P*(·), as shown in Equation (9). Second, to stimulate the framework to achieve efficient convergence on complex images, such as small object-contained images and partial occlusions, we introduce another Loss function (*L*_*comp*_), as shown in Equation (10). Finally, we design a feature transformation loss *L*_*ft*_ to train Hough transfom-based process, as shown in Equation (11). Here, *D* is a discriminant that distinguishes between the ground-truth and the predictions, and *g*_*cls*_ denotes the prediction ground-truth. The proposed framework is programmed in an end-to-end manner.


(8) $$\begin{array}{l} L_{asso}=L_{bce}+L_{comp}+L_{ft} \end{array}$$

(9) $$\begin{array}{l} L_{bce}=\overset{N}{\underset{i=1}{\sum }}\alpha m_{i}\cdot \log\left(p\left(m_{i}\right)\right)+\left(1-\alpha \right)\left(1-m_{i}\right)\cdot \log\left(1-p\left(m_{i}\right)\right) \end{array}$$

(10) $$\begin{array}{l} L_{comp}=1-P\left(m_{i}\right)\log_{2}P\left(m_{i}\right) \end{array}$$

(11) $$\begin{array}{l} L_{ft}=\sum D\left(f_{IP\to BEV}\cdot f_{ip}\left(X\right),g_{cls}\right) \end{array}$$

## 4. Experiment and discussion

In this subsection, we first empirically compare CNNs with Transformers and discuss the results. Next, we empirically assess the effectiveness of the proposed method on two challenging benchmarks and compare it with state-of-the-art methods. Moreover, we show the performance of neural networks on typical challenging tasks.

### 4.1. Experimental settings

#### 4.1.1. Database

We choose two particularly challenging benchmarks to evaluate the proposed model, i.e., the NuScenes dataset (Caesar et al., 2020) and the Argoverse 3D dataset (Chang et al., 2019). They are large-scale datasets in the field of autonomous driving. For data selection, we follow the standard procedures used in most previous studies (Philion and Fidler, 2020; Roddick and Cipolla, 2020). From the NuScenes dataset, we select four categories of maps predicted by images, which contain 14 elements. From the Argoverse 3D dataset, we select eight out of 15 elements for map prediction. Additionally, since both datasets are designed for object detection, and the labels are provided in vectorized and 3D bounding boxes, we regenerate labels to fit the map prediction task. As for technical details, we follow the recent works Philion and Fidler (2020) and Roddick and Cipolla (2020). The main approach we apply is generating annotations for rasterized BEV images via vector label mapping and binary mask generation. The predicted elements consist of Drivable (Dri.), Pedestrian Crossing (Ped.C.), Vehicle (Veh.), Large Vehicle (L.Veh.), Walkway (Wal.), Carpark (Carp.), Car, Truck, Bus, Trailer (Tra.), Construction Vehicle (Con.V.), Pedestrian (Ped.), Motorcycle (Mot.), Bicycle (Bic.), Traffic cone (Tra.C.), and Barrier (Bar.).

#### 4.1.2. Evaluation

To ensure fairness in comparison, we select the Intersection over Union (IoU) score as the main evaluation metric. The IoU evaluation shows the similarity between the element prediction area and the ground truth area, with higher values indicating more accurate predictions.

#### 4.1.3. Implementation

We first pre-train the proposed network with the ImageNet dataset using SGD, with a batch size of 512. Considering that the smaller the input patch size is, the more computationally expensive it is, we choose a patch size of 64 × 64. The number of attention heads is 6, and the number of the transformer blocks is increased to 6 (typically 4). The Adam algorithm is utilized for training, with a weight decay of 0.1 and a batch size of 32.

### 4.2. Ablation study

Our first step is to conduct ablation study on the NuSences dataset (14 elements) to evaluate the effectiveness of two proposed fundamental techniques: Transformer-based feature extraction model (ViT-FE) and the Transformer-based BEV semantic representation generation model (ViT-RG). The main purpose of this study is to assess how well Transformers perform in BEV representation prediction. In most CNN-based studies, ResNet-based networks are used for feature extraction, and networks with a Top-down structure are used for BEV representation generation. Hence, we mix and match the proposed different modules and the leading CNN-based networks.

In the ablation study, we select three leading deep modules for mixing and matching, including a ResNet-50 backbone (R) (He et al., 2016), a ResNet-50 with the FPN structure (R-FPN) (Yu et al., 2022), and an IPM-based Top-down network (IPM) (Deng et al., 2019). We train the above three models using SGD with a momentum of 0.9, a batch size of 32, and a wight decay of 0.1.

Table 1 shows the results of the ablation study, and all of the Abbreviated names are listed above for reference. The results clearly indicate that the proposed ViT-FE and ViT-RG show a considerable improvement in performance as compared to CNN-based models, with an increase of around 6% higher (Mean IoU). These findings highlight the effectiveness of using Transformer-based to predict BEV semantic representation. Specifically, the Transformer-based modules can gradually improve the prediction accuracy for large-scale objects by about 2% and significantly improve it for challenging small-scale objects by about 1% IoU.

**Table 1:** Performance of various design.

**Method**	**Dri**.	**Bar**.	**Ped.C**.	**Wal**.	**Carp**.	**Car**	**Truck**	**Bus**	**Tra**.	**Con.V**.	**Ped**.	**Mot**.	**Bic**.	**Tra.C**.	**Mean**
R + IPM	44.3	2.7	10.6	13.8	13.2	4.7	0.2	2.7	7.4	6.2	0.6	0.6	0.3	2.4	7.9
R-FPN + IPM	53.7	5.1	14.9	16.3	14.7	11.2	5.8	9.7	11.1	8.2	2.4	2.7	2.1	3.9	11.6
ViT-FE + IPM	56.4	7.1	27.3	26.1	15.8	23.6	16.1	20	15.6	11.7	7.1	5.7	7.5	5.4	17.5
R + ViT-RG	55.2	7.3	23.6	24.7	15.2	20.7	12.2	17.2	14.3	10.4	5.8	4.4	6	4.5	15.8
R-FPN + ViT-RG	56.3	10	28	28.5	17.4	23.4	15.7	19.9	15.8	11.7	6.7	5.6	6.9	5.3	17.9
Proposed	61.1	11.9	28.7	32.7	19.2	25.8	17.4	20.6	16.9	12.3	8.3	7.3	9.8	6.6	19.9

A larger value indicates better performance.

**Figure 4:**
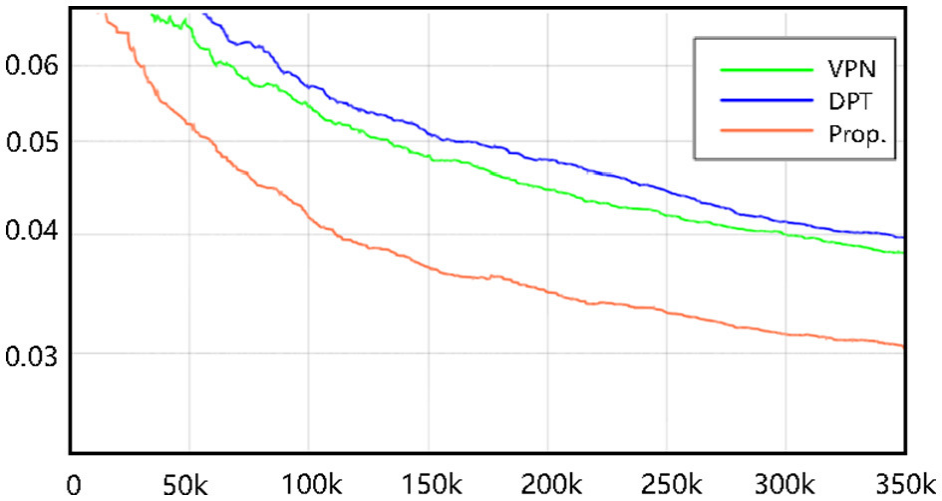
The loss function curves for three methods on the NuScenes.

Ensuring stable training is important for neural networks, especially for Transformer-based networks. We propose a new framework based purely on Transformers. To evaluate the training effect of the proposed neural networks, we conduct empirical experiments on a challenging benchmark (NuScenes). As depicted in Figure 4, our method achieves comparable training performance to other CNN-based methods. We suppose that the proposed Transformer-based neural networks can be generalized for the BEV semantic prediction task. Stable training serves as the basis for further improving the performance of Transformer-based methods.

### 4.3. Main results and comparison to state-of-the-art works

In this subsection, we present a comparison of the proposed model with three recent works, including a View Parsing Network (VPN, published in IEEE RAL, 2020) (Pan et al., 2020), a Top-down network with transformer layer (DPT, published in CVPR 2020) (Roddick and Cipolla, 2020), and Lift-Splat-Shoot network (L-S-S, published in ECCV 2020) (Philion and Fidler, 2020). The CNN-based baseline [R-FPN (Yu et al., 2022) + IPM (Deng et al., 2019)] is also shown for reference. It should be noted that the sub-datasets chosen for each study are not identical due to the large scale of autonomous driving datasets. To ensure a fair comparison, we follow the part of the results reported by the DPT, and then we train and test the L-S-S using the same experimental setup.

#### 4.3.1. Main results

**Figure 5:**
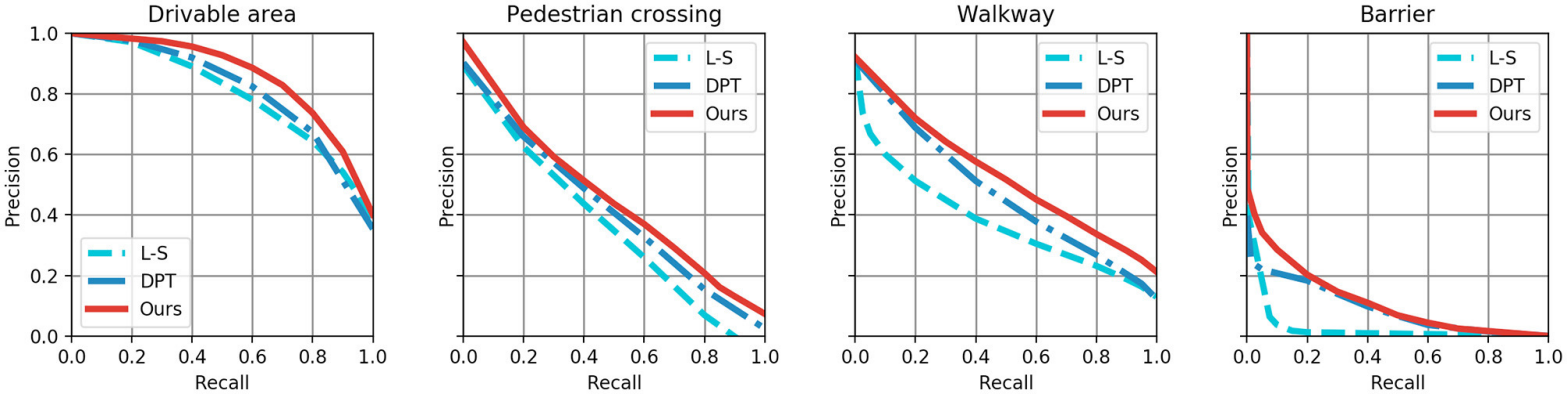
Precision-recall tradeoff on the NuScenes dataset.

The NuScenes dataset contains a greater variety of objects than the Argoverse 3D dataset, making it more challenging. As demonstrated in Table 2 our proposed network achieved a significant improvement in the Mean IoU metric, with 0.8% higher than the DPT, 1.4% higher than the L-S-S, and 2.4% higher than the VPN. Table 3 shows that the proposed network attains the leading performance on the Argoverse 3D dataset. Specifically, the proposed network exhibits further improvements in the prediction rate of large-scale objects by about 2% in the prediction rate of large-scale objects. The main result is breaking through the bottleneck of small-scale object prediction. In comparison, the prediction accuracy based on the CNN networks remains essentially unchanged. Figure 5 shows the Precision-recall curves of four interesting elements selected from the NuScenes dataset. The closer these curves are to the upper right, the better the models prediction performance for positive samples.

**Table 2:** Main results (IoU) on the NuScenes dataset.

**Method**	**Dri**.	**Bar**.	**Ped.C**.	**Wal**.	**Carp**.	**Car**	**Truck**	**Bus**	**Tra**.	**Con.V**.	**Ped**.	**Mot**.	**Bic**.	**Tra.C**.	**Mean**
R-FPN + IPM	53.7	5.1	14.9	16.3	14.7	11.2	5.8	9.7	11.1	8.2	2.4	2.7	2.1	3.9	11.6
VPN (Pan et al., 2020)	58	10.8	27.3	29.4	12.9	25.5	17.3	20	16.6	4.9	7.1	5.6	4.4	4.6	17.5
L-S-S (Philion and Fidler, 2020)	60.2	10.9	26.6	31.1	17.6	24.2	16.5	20.3	15.4	10.8	7.5	5.8	7.2	5.9	18.5
DPT (Roddick and Cipolla, 2020)	60.4	8.1	28	31	18.4	24.7	16.8	20.8	16.6	12.3	8.2	7	9.4	5.7	19.1
Proposed	61.1	11.9	28.7	32.7	19.2	25.8	17.4	20.6	16.9	12.3	8.3	7.3	9.8	6.6	19.9

A larger value indicates better performance.

**Table 3:** Main results (IoU) on the Argoverse 3D dataset.

**Method**	**Dri**.	**Bus**	**Tra**.	**L.Veh**.	**Ped**.	**Mot**.	**Bic**.	**Veh**.	**Mean**
R-FPN + IPM	54.2	5.2	0.3	8.5	2.7	0.8	0.2	15.8	11
VPN (Pan et al., 2020)	64.9	3	0.4	9.7	6.2	1.9	0.9	23.9	13.9
L-S-S (Philion and Fidler, 2020)	65.2	13.7	1.8	11.7	8	5.7	3.4	30.8	17.5
DPT (Roddick and Cipolla, 2020)	65.4	11	0.7	11.1	7.4	5.7	3.6	31.4	17
Proposed	66	17.5	2.9	13.8	8.6	6.4	4.2	33.7	19.1

A larger value indicates better performance.

#### 4.3.2. Discussion

Through an extensive examination of our experimental results, several key insights have been gleaned, reaffirming the innovative nature and potential of the method. First, Transformer-based networks substantially improved the prediction of BEV semantic features. This represents a significant stride forward compared to traditional methods, suggesting that Transformers hold great promise in advancing BEV mapping capabilities. This marked performance enhancement demonstrates the effectiveness of Transformer-based approaches. It provides a valuable reference point for future research endeavors, opening up new avenues for exploration and innovation. Second, our proposed Transformer-based feature extractor demonstrated a superior ability to extract finer features than CNN-based networks. This superiority is particularly significant in predicting small-scale objects. In this task, the extraction of intricate details is of utmost importance. This underlines the capacity of Transformer-based models to outperform their CNN counterparts in tasks that require a keen discernment of finer details, thus broadening their potential applications in related fields such as object detection and recognition. Last, our unique contribution is the introduction of a Transformer-based feature generator capable of outputting pixel points instead of per-class probability. This novel approach has exhibited superior performance compared to traditional Top-down networks. By moving from per-class possibilities to pixel point outputs, the proposed model offers a more nuanced and detailed understanding of the input image, essential for complex image generation tasks like BEV mapping. It also presents a more versatile and granular output format that can be more readily adapted to various applications. These observations demonstrate the superiority and innovation of our proposed Transformer-based approach to BEV semantic feature extraction and generation. This research has not only bridged a significant gap in the field but also paved the way for further advancements and applications of Transformer-based models in the broader domain of image processing and analysis.

### 4.4. Performance on challenging scenarios

This paper aims to making contribution to the discussion of global-local spatial relationship learning, which is better at multi-class and multi-scale element prediction. To further show the performance of the proposed method on challenging tasks, we evaluate its performance using challenging samples, i.e., complex lanes, small-scale pedestrian, dark environments, two key traffic signals, and multi-class element.

**Figure 6:**
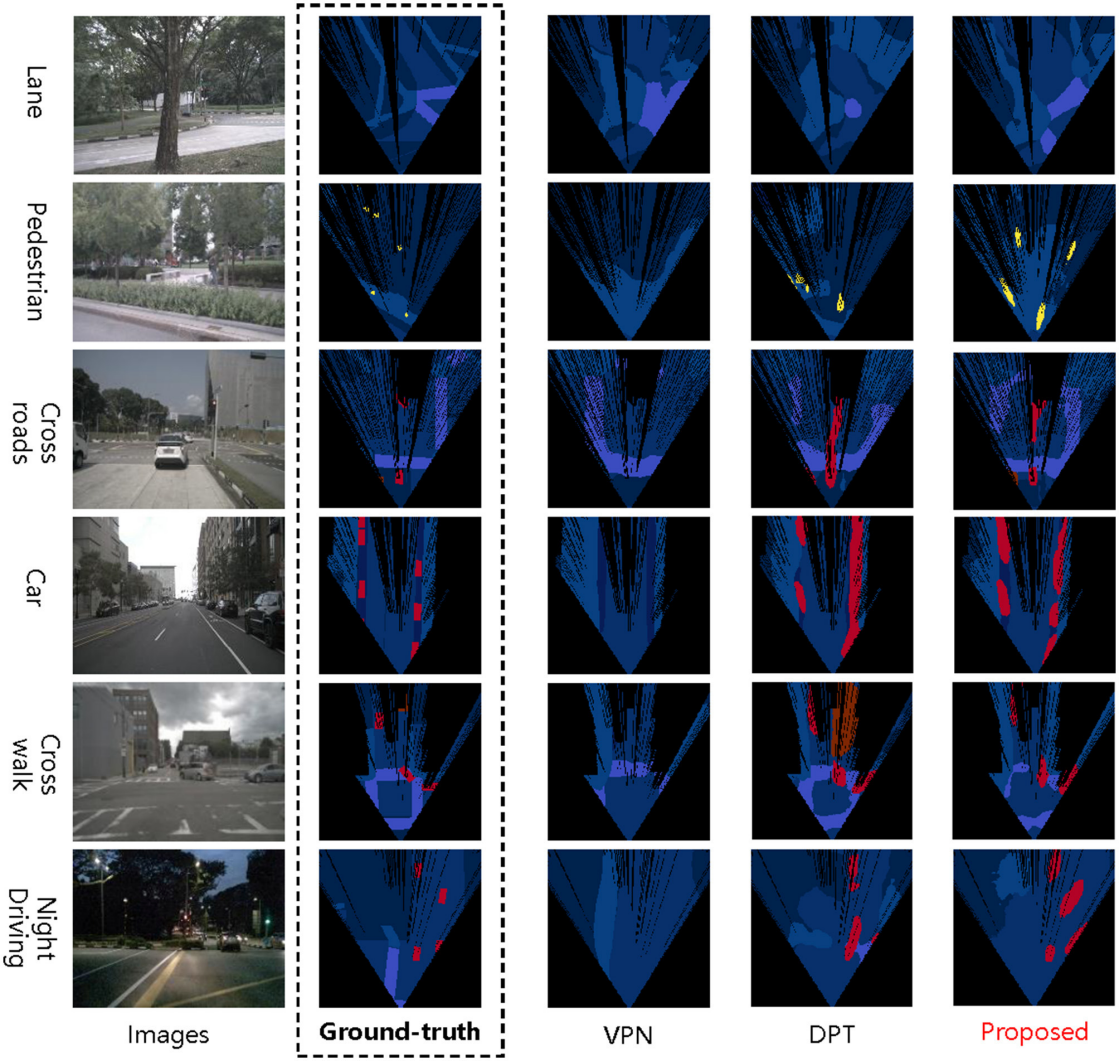
Qualitative results on the NuScenes dataset. For a fair comparison, we follow the color scheme utilized in the DPT. We evaluate methods on six challenging scenarios and compare the proposed method with two baselines.

Figure 6 shows the qualitative results on the NuScenes dataset. Two state-of-the-art methods are introduced for comparison. The key conclusions are as follows. (1) The proposed method predicts BEV semantic representation that closely matches ground-truth labels. (2) The proposed method can effectively perceive more detailed features, such as vehicle contours, subtle changes in lane lines, and small-sized pedestrians. For example, the VPN fails to predict small-size elements like pedestrians, and the DPT only predicts elements that are close to the camera, while the proposed method works well. (3) The proposed method can achieve state-of-the-art performance in complex field situations, including illuminant-changed scenarios and occlusion. For example, in the night driving, the prediction result of the VPN does not contain vehicles, and the DPT can only predict parts of vehicles. In comparison, the proposed method can still accurately predict the number and location of vehicles in occlusion scenarios.

**Figure 7:**
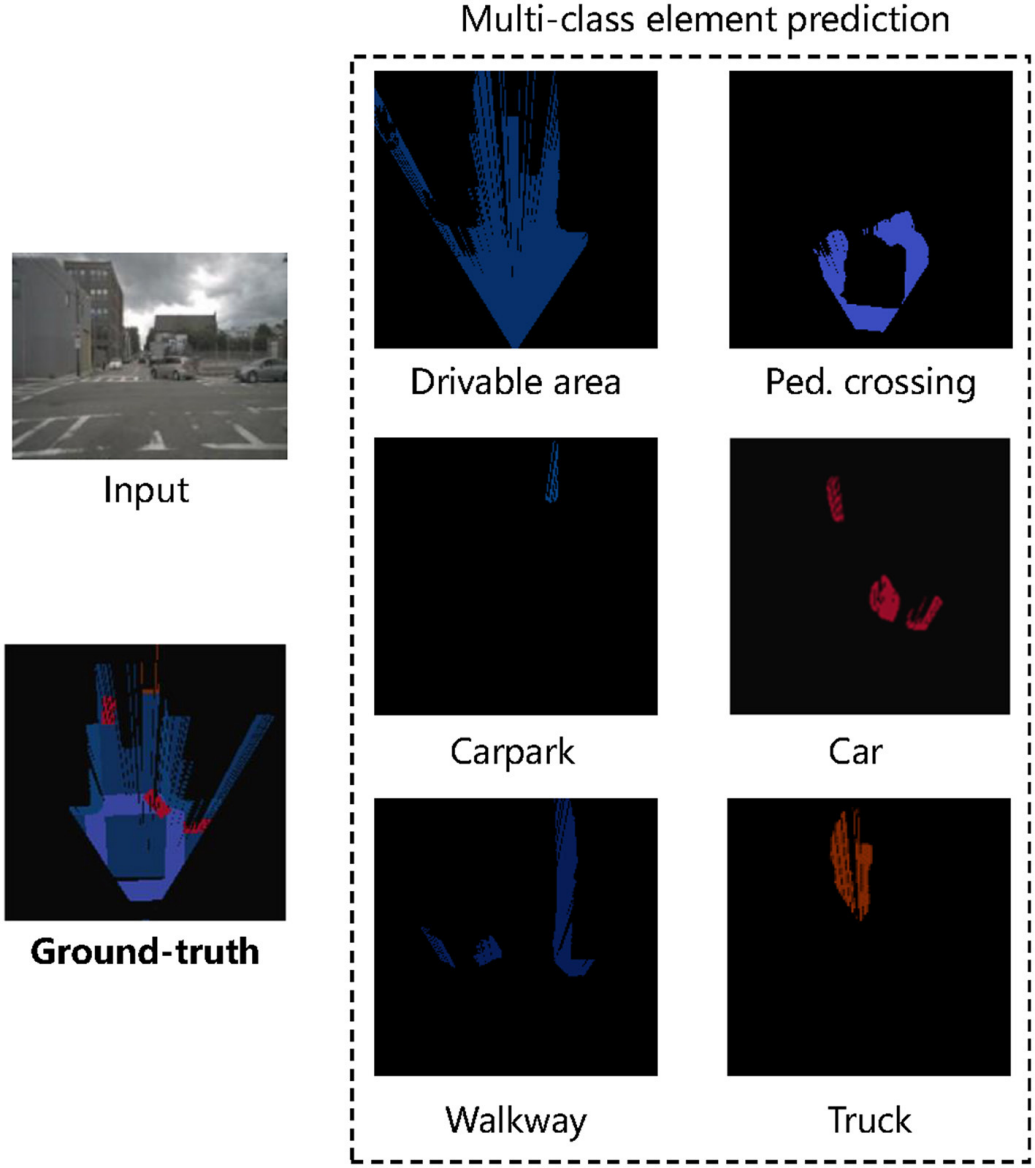
Visualization of the multi-class element prediction results on the NuScenes. An autonomous vehicle predicts a BEV representation with six elements in a cloudy scene. The BEV representation of the six elements is present separately.

Furthermore, we test our proposed method by examining its performance on multi-class element prediction, as shown in Figure 7. We deployed the technique to generate Bird's Eye View (BEV) semantic representations for each element in scenarios that pose significant challenges, such as occlusion, the presence of multi-class/multi-scale objects, and dim illumination. The results have been highly encouraging, demonstrating that our model can accurately predict the position and shape of each element. These positive results confirm our method's effectiveness and point toward its high computational efficiency and scalability, particularly in large-scale environments. Despite the complexity introduced by multi-class/multi-scale objects and conditions like occlusion and dim lighting, the model maintains an impressive performance. This attests to the model's robustness and adaptability in diverse and challenging situations. Notably, the computational efficiency of our method does not compromise its scalability. Our model can seamlessly handle an increased number of classes or a larger scale of images without requiring a proportional increase in computational resources. This computational scalability is crucial for real-world applications where the model might need to operate in vast and complex environments. This capability could be highly beneficial in numerous practical applications, from autonomous navigation systems to robotics, which requires a nuanced understanding of their surroundings.

## 5. Conclusions

The paper presents novel neural networks powered by Transformers for BEV representation prediction, which is substantially different from CNN-based networks which are commonly reported in existing works. Our method focuses on map generation through image-plane feature extraction and transformation, without the use of convolution and pooling layers. In this way, per-class element prediction and BEV map generation are implemented through an end-to-end framework. Results demonstrate strong performance on two large-scale benchmarks, i.e., the NuScenes dataset and the Argoverse 3D dataset. The model attains greater accuracy improvement for large-size object prediction (about 2 % IoU) and a breakthrough for small-scale object prediction (about 1 % IoU). Furthermore, the proposed method shows a leading performance in challenging scenarios.

In the future, we will study (1) train Transformer-based networks with less data, (2) memorize more distant global clues, and (3) build a Transformer-based temporal framework. We argue that the boost in performance of BEV representation prediction depends on spatiotemporal relationship mining, and balancing between data-driven approaches and performance-boosting techniques is key for deep learning.

## Data availability statement

The original contributions presented in the study are included in the article/supplementary material, further inquiries can be directed to the corresponding author.

## Author contributions

JY: conceptualization, methodology, software, and writing—original draft. WZ: software, validation, formal analysis, and writing—original draft. YC: conceptualization, supervision, writing—review and editing, and funding acquisition. YZ: data curation and validation. RH: project administration. All authors contributed to the article and approved the submitted version.
